# Modified Constraint-Induced Movement Therapy at Home—Is It Possible? Families and Children’s Experience

**DOI:** 10.3390/children7110248

**Published:** 2020-11-22

**Authors:** Rocío Palomo-Carrión, Helena Romay-Barrero, Rita-Pilar Romero-Galisteo, Elena Pinero-Pinto, Purificación López-Muñoz, Inés Martínez-Galán

**Affiliations:** 1 Department of Nursery, Physiotherapy and Occupational Therapy, Faculty of Physiotherapy and Nursing, University of Castilla-La Mancha, 45500 Toledo, Spain; Rocio.Palomo@uclm.es (R.P.-C.); Purificacion.Lopez@uclm.es (P.L.-M.); Ines.Martinez@uclm.es (I.M.-G.); 2 Group of Physiotherapy Research of Toledo (GIFTO), 45500 Toledo, Spain; 3 Department of Physiotherapy, Faculty of Health Sciences, University of Málaga, 29071 Málaga, Spain; 4 Department of Physical Therapy, Faculty of Nursing, Physiotherapy and Podiatry, University of Sevilla, 41009 Sevilla, Spain

**Keywords:** constraint-induced movement therapy, child, experience, family, home, infantile hemiplegia, qualitative, self-determination, upper extremity

## Abstract

Modified constraint-induced movement therapy (mCIMT) is efficient at improving upper limb non-use. The experiences of families and children with mCIMT could allow researchers to understand how it influences their day-to-day life and to improve the function of the affected upper limb without altering family life and avoiding frustration. In this qualitative study, we aimed to collect the experiences of parents and their children (aged 4–8 years) who did mCIMT at home regarding the application of low-intensity modified constraint-induced movement therapy to improve the affected upper limb functionality in infantile hemiplegia with moderate manual ability. Individual semi-structured interviews were performed to obtain insights into their experience with mCIMT. The experiences of parents and children were described in thematic sections. Eight children with hemiplegia (six years, standard deviation, SD: 1.77) and their parents were asked about their experiences after applying 50 h of mCIMT at home. Three main themes emerged from the children’s interview data: (1) the experience of wearing the containment in the modified constraint-induced movement therapy (CIMT) intervention, (2) the reaction to performing the therapy at home with his/her family, and (3) learning of the affected upper limb. In the parents’ interview data, there were two main themes: (1) the difficulty of executing an intensive therapy protocol (mCIMT: 50 h) at home and (2) the feeling of not wanting to finish the intervention. The experiences of the parents and their children regarding mCIMT allowed us to understand the facilitators and barriers that affect the execution of mCIMT at home, and this understanding allows us to improve its future application.

## 1. Introduction

Constraint-induced movement therapy (CIMT) is a short-term, intensive treatment to improve the affected upper limb (UL) functionality in children with hemiplegia [1]. CIMT includes containment of the unaffected UL, intensive training of the affected UL, and the use of behavioral methods to encourage children’s effective participation in the intervention process [2]. In the original protocol, the unaffected UL is constrained for 90% of the daily time, during which the individual is awake, and the therapy is performed 6 h per day for 2–3 weeks, using unimanual activities progressively graded in increasing difficulty, targeting the child’s most relevant limiting factor that hampers functional use of the affected hand [2,3].

The high time requirement of the intervention makes its execution complex in pediatrics. Thus, the number of weeks of therapy began to increase and the daily time was reduced; this modification of CIMT is called modified constraint-induced movement therapy (mCIMT) [4,5,6]. There has been considerable variation in the total daily dose applied, as well as in the proportion of “direct” intervention (delivered by therapists) and “indirect” therapy (through the use of home/preschool or school programs applying mCIMT) [7,8]. Due to the lower daily dose, mCIMT is more consistent with the philosophy of family-centered interventions, since it allows parents to remain in the role of “expert” caregiver for their child [9]. However, home interventions guided by family members could interfere with family dynamics, presenting the child with stressful experiences as a result of having to deal with the demands of activities using only the affected arm, as well as the impact on the structure and flow of the daily routine. In addition, other stressors that can alter family dynamics and lead to frustration in the child must be taken into account: protocols that cannot be adapted to daily routines (school, daily activities, etc.), containment, type of activities, repetition, lack of motivation, etc. [7,8]. Therefore, if parents were trained by a therapist on the proper management of the intervention, considering their worries and needs, the intervention could be tailored to their child’s tolerance and structure their routines to incorporate the containment and the accompanying therapy time. Parents can select activities to be included in the therapy that are meaningful and motivating for themselves and their children, which results in a greater adherence of both parties (parents and children) to the treatment [8].

Most studies on mCIMT show its efficacy at improving affected UL non-use [6,10,11,12]. Different investigations have analyzed the experiences of children, caregivers, and parents in CIMT application [13,14,15,16], although none have collected the parents’ and children’s perceptions or experiences of the application of CIMT at home. However, it is necessary to collect the perspectives of children and their parents when the intervention is carried out at home and determine the impact on their day-to-day life, to improve the function of the affected UL without altering family life and avoiding frustration. It would be important to know the experiences and feelings of the children and parents during the therapy execution process. In this way, complications could be reduced, obtaining greater benefits. For this reason, the perceptions of the agents involved in the intervention should be recorded to improve their involvement and encourage their adherence.

Thus, in this study, we aimed to collect the experiences of parents and their children (aged 4–8 years) who did mCIMT at home in the application of low-intensity (<3 h per day) modified constraint-induced movement therapy to improve the affected UL functionality in infantile hemiplegia with moderate manual ability [17].

## 2. Materials and Methods

In our study, we investigated eight children with infantile hemiplegia with moderate manual ability, who were part of a study on the application of low-intensity modified constraint-induced movement therapy (mCIMT) to improve the functionality of the affected UL. The children performed a low amount of mCIMT (50 h: 2 h per day for 5 weeks). McConnell et al. [18] applied mCIMT for two weeks with two hours per day in a clinical setting, which was designed for children aged 8–15 years. The children executed a total of 20 h of mCIMT with functional changes. In our study, we suggested a total of 50 h with the same distribution per day according to the abovementioned study, but with an additional 30 h, since the participants in our study were younger than those in McConnell et al. [18] and they would thus need a greater amount of time due to the lack of attention and to the fact that the therapy was performed at home, as the children and their families would need more time to obtain significant outcomes. We decided to assess the children at week 2 of treatment (20 h) to verify whether the changes would be the same at week 5 (after treatment with a total time of 50 h). Measuring at 5 weeks made it easier to divide the total time (50 h: 10 h per week). We determined that two hours per day was feasible. The parents were asked if they could complete this therapy time, and they responded that, despite requiring some effort, the 5-week period would be optimal, with the same dose per day and not excessively lengthened in time.

The methodology of intervention was based on the following:

(1) Home-based family training: Prior to the beginning of the study, an informative meeting was held with the parents, in which all the details of the study were explained to them and a program of unimanual activities was given to them to be executed with the affected upper limb every treatment week. At the meeting, they were taught how to perform the unaffected hand containment with the bandage. In addition, the therapist demonstrated the different tasks proposed, showing the necessary materials (or the possibility of using others), position of the child, position of the parents, and modification of the activity with other possible ones that motivate the child. Before the intervention, an online meeting was held with each family to review the activities of the first week, address doubts and adapt them to the child’s preferences, which had previously been sent by the parents via email, with the aim of encouraging the child’s motivation.

(2) Child motivation: Motivation was induced through activities designed according to the children’s preferences to avoid frustration, carried out through a story that the parents told them, where the child could dress up. In addition, the parents were trained in weekly follow-ups to encourage positive feedback using smile stickers when the therapy was completed. These stickers were put on a weekly record and, if the 5 days of the 5 weeks of intervention had a smile sticker, the child would receive an “achievement medal” along with a diploma for having performed all the exercises.

(3) Intervention protocol: The study was carried out over a period of 5 weeks of treatment, containing the unaffected upper limb for 2 h per day (not continuously) from Monday to Friday. The children were requested to perform the structured activities for two non-consecutive hours, with the aim of increasing the adherence and avoiding great physical effort. The parents were advised to set aside one hour in the early afternoon and another hour in the late afternoon to ensure that the child was attentive, frustration-free, and effort-tolerant. The families were also instructed to run a full hour, repeating the activity and designing a story, in which the child was the protagonist, and the activity was an enjoyable game to complete. Each of the proposed hours could be divided into periods of 30 min to improve the child’s attention and motivation, in case the child was tired during the treatment. When dealing with children from 4 to 8 years old, attention is greater and maintained for a longer time, which allows establishing a continuity of 1 h of intervention. The intervention was carried out by a member of the family at home. The activities were programmed to practice different movements that were limited in the affected upper limb: shoulder flexion, elbow extension, forearm supination, wrist extension, and grasp. Each activity was repeated for around 15 min to obtain a lesson about a functional strategy to use in their usual activities. Each intervention hour was completed with four activities, which were repeated in the second hour to increase the movement learning and the possibility to modify and obtain new functional strategies to perform the activity. The same activities were performed every week (first and second hours from Monday to Friday) to improve the movement (shoulder flexion, forearm supination, elbow extension, wrist extension, and grasp–release). The family member in charge of carrying out the therapy with their child had to provide them with the objects intended for the hour of the session, place the objects or games in a suitable position so that the child could pick them up or throw them, etc., guide the movement if the child did not use an adequate trajectory, stimulate the child to change the functional strategy if the one used was not suitable, control the child’s position and avoid compensation, motivate the child in the execution of the task, and reward his success in achieving it.

(4) Weekly follow-up of the intervention to promote adherence: In this follow-up, the parents commented on their complications, their fears, and their doubts concerning the exercises and the unaffected containment, and the therapist was in charge of supporting them, facilitating the execution of the activities by modifying them for others following the child’s preferences and ensuring the safety of the children, providing necessary changes, and making sure that they were executing the activities properly. The therapist and the families met in the assessment center or home setting every week to review the activities and make adjustments if necessary. This meeting was held on Saturdays when they had finished the intervention of the week and thus assessed the difficulties presented during the same week and the incorporation of new activities according to what was appreciated and the needs observed. In addition, in that meeting, the new ludic activities chosen by the child were practiced, inducing their correct execution, as well as greater confidence of the parents in the execution. Moreover, they were given the possibility of contacting the therapist via email or telephone, at any time, to address any doubt or urgent complication that would affect the execution of the therapy. The therapeutic goal was always to avoid the frustration of the child, the insecurity of the parents, and to introduce satisfaction and feedback—children–parents–therapist.

Details of the study have been previously published [17].

The inclusion criteria were as follows: medical diagnosis of left/right congenital infantile hemiplegia, age between 4 and 8 years, lack of activity of the affected UL, exceeded 10° extension in the metacarpophalangeal and interphalangeal joint, completed the 20° extension of the wrist of the affected UL, adequate cognitive development to understand the verbal orders given for the execution of the tasks and cooperation in their execution. The exclusion criteria were as follows: visual problems that prevented the individual from carrying out the intervention, significant additional balance disturbances that put the child at risk of falling as a consequence of having the unaffected UL contained, presenting uncontrolled epilepsy, and having received botulinum toxin within 6 months prior to the intervention.

### 2.1. Data Collection

Each interview was carried out individually with an average duration of 20 min in the home setting. The questions asked in the interview were designed based on the concerns presented during the meeting with the families and the training with the parents, in addition to those reported in different investigations [13,14,15,16]. It was decided to carry out the interview with the family member who had conducted the intervention, since they would have a more reliable perspective towards its development and difficulties, thus, highlighting the complications that were perceived according to their own experience. Interviews were individually conducted with three main open-ended questions for the parent (mother/father or both) who carried out the intervention with the child: 1. What was your experience from this therapy? 2. What was your experience during the mCIMT application? 3. Were your expectations met? The children were asked one question: How did you feel during the application of this therapy? Specific questions were asked to include important aspects that could not be considered by the parents or children during the interview.

This method of data collection was chosen to give the participants a chance to reflect individually on their experience. The interview questions were focused on how the parent who conducted the intervention and the child who did the mCIMT perceived the intervention execution at home, the programming of activities, the use of containment, the experience of using the affected hand, complications in their execution at home, whether it was difficult to include the intervention into daily routines, the effort involved in participating, any differences the child noticed in the follow-up, etc. The main questions are presented in Table 1.

### 2.2. Data Analysis

The responses obtained from the interviews were collected by two researchers, who digitally recorded the interviews and transcribed the recording verbatim for analysis. They checked the accuracy of the transcripts against the digital recordings, corrected errors, and changed all identifying information to ensure confidentiality. The methodology of hermeneutic phenomenological data analysis was applied [19]. This methodology is based on the way things appear to us through experience or in our consciousness, where the researcher aims to provide a rich textured description of lived experience. It is focused on people’s perceptions of the world that they live in and what it means to them, i.e., people’s lived experience. As a qualitative method, it focuses on human experience as a topic in its own right. It is concerned with meaning and the way in which meaning arises in experience [19]. The procedure of phenomenological data analysis was conducted as follows: at first, each transcript was read several times to get a general impression of the interviews. Subsequently, ‘significant statements’ about the experiences of the parents and children with mCIMT were identified and highlighted. Thereafter, the ‘significant statements’ were combined to create themes. Finally, the experiences of the parents who conducted the intervention and the children who did the mCIMT were described in thematic sections, by rewriting them [19]. The children and their parents were labeled with numbers (1–8) in order to maintain their anonymity.

## 3. Results

All eight children allocated to mCIMT completed the intervention. Table 2 shows the characteristics of the participants: children who did the mCIMT and parents who conducted the intervention.

Three main themes emerged from the children’s interview data: their experience of wearing the containment in the mCIMT, their reaction to performing the therapy at home with their parents, and their learning of the affected upper limb use. From the data of the interview with the parent who conducted the intervention, two main themes were obtained: the difficulty of executing an intensive therapy protocol (mCIMT: 50 h) at home and the feeling of not wanting to finish the intervention.

### 3.1. Children’s Experience

Experience of Wearing the Containment (Partial Containment Using a Bandage) in the Modified CIMT, Children’s Reaction to Performing the Therapy at Home with their Parents and Learning from the Affected Upper Limb.

Below are the responses collected from the eight children about their feeling of wearing containment in their unaffected hand. What did they perceive? Did they feel frustration? Was it easy for them to adapt to having their skillful hand covered? In addition, carrying out an intervention at home can have great advantages in organizing the execution time and finding space to do the activities; however, the involvement of the parent who conducted the intervention can be favorable or unfavorable, depending on how the intervention was directed and the acceptance of both (parent and child) towards it. Lastly, learning to use something unknown is challenging (affected upper limb), since one must first discover its existence. How was this discovery of the affected hand in children?

#### Children’s Testimony

Child 1 (4 years old). “I did not want to carry that bandage in my good hand to use the other hand, I didn’t want to use that hand because it didn’t work. The worst was to use the other hand”. “Playing the games at home was a lot of fun because I spent a lot of time with Mom and enjoyed our stories, although I didn’t like using the other hand”. “Now I can play with my dolls, comb them, rock them… I’m very happy”. 

Child 2 (5 years old). “I liked carrying the bandage in my hand because I could use the other hand in fun games”. “I realized that my house was a magical place full of adventures and that my mother was very funny”. “And, now, my hand is no longer asleep, sometimes she wakes up unannounced and helps me, I named it as sleeping hand”. 

Child 3 (5 years old). “I didn’t want to wear the bandage, it was weird to have it on and I had to use my other hand even though I didn’t like it, but the activities were fun and I liked doing them with the hand that I didn’t use”. “Doing the exercises with my mother was a lot of fun; now we’re a team and we’ve discovered places at home, like the Fire Dragon Lagoon. I want to do it again”. “I can play with my tools and fix the cars I had broken, using my hands. I love it. I couldn’t do this before”. 

Child 4 (5 years old). “Wearing the bandage was fun because I decorated it and I turned into a superhero who discovered his powers using the other hand in fun games, and because of that, I’d give my mother the bandage myself to put it on my good hand”. “Having a magic box of toys and being a magician, Mom’s magician, was a lot of fun; we had a great time”. “Now, I can go up and down the slide using my two hands with my friends”. 

Child 5 (7 years old). “When my mother put the bandage on me I cried because I didn’t want to use my other hand. I never use it because I don’t do anything right with it, but it was fun to play the games”. “My mother always waited for me with the adventure story to start the story and do the exercises; every day I was a new character and I dressed up. Doing the activities with her was great…”. “Sometimes, I didn’t want to use my hand because I was so clumsy, but now I use it to do a lot of things without realizing it”. 

Child 6 (6 years old). “I didn’t like wearing the bandage, but I realized that I could use my other hand if I tried”. “Although I didn’t like wearing the bandage, playing the games with my mother was a lot of fun; we spent a lot of time together and laughing, although I didn’t play the game very well”. “I know I have two hands and that I can use them together”. 

Child 7 (8 years old). “My father explained to me that I had to wear the bandage for the other hand to work better, and I enjoyed playing with him”. “We built our own playbox and every corner of the house had a riddle to solve. My father asked me and I had to solve the riddle. It was very good”. “Sometimes, I’m dressing with both hands… it’s a weird feeling; now that hand is better, it helps me”. 

Child 8 (8 years old). “I cried a lot when I had my good hand covered, I didn’t want to use the other hand, because I didn’t know how to use it, but I decorated my bandage and it was fun to put it on and imagine that my hand could disappear and the other hand would appear”. “to be at home was very good”. The games were chosen by me and Mom and we invented a story every day, I didn’t want it to end”. “I’ve learned to use my hand better and not to get angry if sometimes it doesn’t work well, because I can do it all with one, but if the other helps me I can do things, like brushing my teeth. I just couldn’t do that before”. 

### 3.2. Parents’ Experience 

Difficulty of Executing an Intensive Therapy Protocol (mCIMT = 50 h) at Home and the Feeling of not Wanting to Finish the Intervention

Executing an intervention at home with the participation of the parent who conducted the intervention must have different considerations, including being able to adapt the protocol to their needs and worries. What feelings do parents have about participating in the therapy within their natural environment (home)? When an intervention directed at home by the parents produces satisfaction, there is a feeling of not wanting to leave it, for fear of losing the benefits obtained. What feelings do parents have when the intervention ends?

#### Testimony of the Parents Who Conducted the Intervention

Mother of Child 1. “At first, I was afraid; I thought that being so young (4 years old) she would not adapt… But I was surprised… we were able to complete all hours inside the house thanks to the weekly follow-up.” “I didn’t want to finish the therapy… my daughter couldn’t play with her doll and after the intervention, I saw her cry the first time she picked her up to give her doll a bottle. A sense of happiness”.

Mother of Child 2. “I was scared about the number of hours we had to do, but when we were doing the therapy, time went too fast… Having a weekly accompaniment with the therapist gave me a lot of peace of mind”. “When the therapy ends you feel like it’s not enough; it has to go on because you’re really afraid that you’ll lose everything you never thought he’d get”.

Mother of Child 3. “I never thought that I would be able to guide my son in the activities and be able to make the times at home because I found it too… But we completed all the times, and sometimes we forgot and the time passed… it was nice to share this intervention together”. “I didn’t want it to end because I realized what my son was capable of doing that I wasn’t aware of before and I was afraid he’d lose that”.

Mother of Child 4. “I was always told it was a very exhausting therapy, and that it was very difficult to find the time to do it… I was scared because doing it at home with my other children would be a great stress… It was really difficult at first to coordinate ourselves and understand how to do everything… then we went too fast”. “I didn’t want our adventure to end. We had built a magic team and the hand he never used was the protagonist, and it didn’t matter how he did it, but that’s what I didn’t know until then; now I’m satisfied with everything that’s been done”.

Mother of Child 5. “The situation at home was not easy. Each of us has a schedule, school activities… besides that, I’m not a therapist, and doing something like this with my child… it caused me anxiety… At first it was difficult, but because my child didn’t want to wear the bandage and she didn’t use her affected hand… I was crying, but having the weekly follow-up gave me a lot of help to continue; otherwise, I don’t know what would have happened… The beginning is difficult…” “I discovered the limitations my daughter had because, until then, she had done it all with her healthy hand, and I appreciated everything she achieved at the end of the therapy. I was very pleased to perform mCIMT, because we have created a very positive feedback and now she is no longer frustrated”.

Mother of Child 6. “I found it impossible to do anything with my daughter at home… it was really complex to work with her, let alone that amount of hours. But the follow-up for the distribution of tasks, on two occasions, allowed us to perform the intervention in the early afternoon; we played stories and, after dinner, we had to look for the lost objects… I laugh when I recall it… It’s a very viable therapy to do at home”. “The first week I asked the therapist if it was normal to want it to end already… because I thought I was doing it wrong as a guide. But then I’d tell him, ‘is it over?’ I don’t want it to end because it helped me know my son’s needs and I want to help him”.

Father of Child 7. “What overwhelmed me the most was when I was told that therapy had to be done at home, but finally the protocol was very feasible to introduce it into the home and we were able to adapt it to our routine. What’s more, now we don’t want to leave home because we have found the therapy that allows us to learn in the environment in which we live… How hard I saw it and how much I’ve learned… Thanks to the training and follow-up care received from the therapist made me more confident and I wasn’t afraid. I just felt satisfaction”. “When the therapy finishes you feel empty; you need that time to be with your child, to have fun, to see that his hand works and that those achievements are not going to disappear. I refused to finish… but, I’m sure we’ll do it again, it’s been wonderful, despite the amount of time consumed”.

Mother of Child 8. “My son had a really hard time being with me. I didn’t want to imagine the time to do therapy… and with so many hours… incorporated into the day-to-day life… with everything you have to do… Well, I realized that if you get organized, there’s time for everything. What’s more, when you finish doing so you miss that time because it is a time of thrill that, thanks to the training received, we were able to adjust very well”. “For me, I love doing mCIMT with my son; I have learned so much… in the end it was the therapy what, in less time, has given us everything. It was the best thing that could have happened to us and now we are looking forward to being told that in the future it can be repeated”.

**Figure 1:**
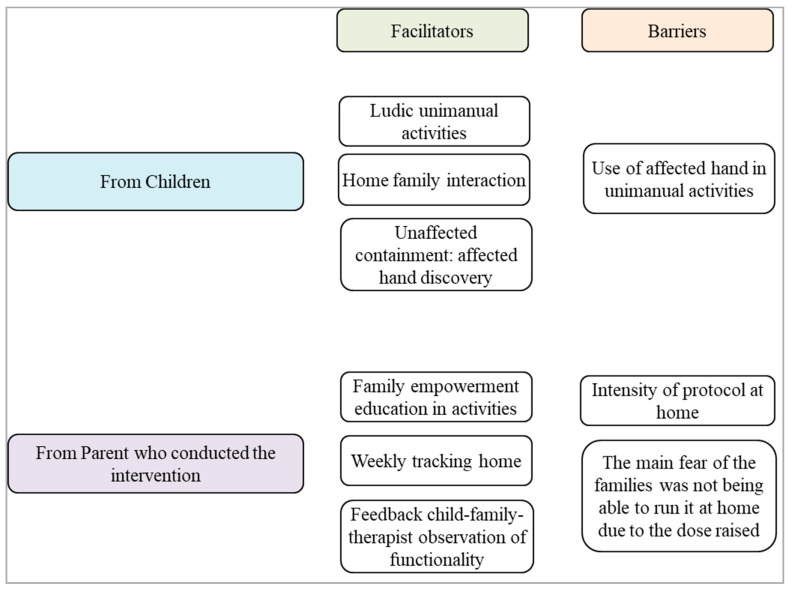
Facilitators and barriers for the children and parents (who conducted the intervention) after applying mCIMT at home.

Figure 1 shows the facilitators and barriers for the children and parents (who conducted the intervention) after applying mCIMT at home.

## 4. Discussion

mCIMT increases the quality of movement and spontaneous use in the affected UL and induces changes in daily routine due to the participation of the affected UL, which results in improvements in the child’s autonomy and self-efficiency [20,21]. Regarding the use of containment, the parents who conducted the mCIMT did not mention that it was difficult to perform the activities with the containment on the unaffected hand. At first, most of the children rejected the containment they carried. The rejection occurred when they had to use their affected hand to do the activities. They surprisingly called their affected hand “the other hand” or “the bad hand” sometimes, which should be reduced to decrease their refusal to use it. Poulsen et al. [22] studied the discomfort of performing bimanual tasks in children with hemiplegia, showing that the use of the affected UL results in rejection, due to the limitations of the affected segment, since children could rely on an extrinsic motivation to do the task [22]. That is, children aim to obtain a rewarding result from the activity through fun and play, with a reward that is appropriate to their goals, and sometimes they receive negative feedback from using their affected hand [22,23]. This could be due to alterations in motor functions and little motivation in the use of the affected hand due to “development disregard”, which is a consequence of the injury produced in the affected hemisphere [24,25]. Therefore, what makes them uncomfortable is not the use of the containment, but the use of their affected hand, which is clumsy, since most of these children have not had previous experience of use with it. According to Gordon et al. [7], the non-use of the UL in children with hemiplegia occurs during a period of their development, when acquisitions have not yet emerged. Thus, while adults have experienced using both hands prior to the brain injury, the same is not true for children, who do not experience successful use of the affected UL, which impacts the development of their bimanual functioning repertoire and results in a phenomenon that is child-specific, defined as developmental disregard [7].

However, when asked about learning from their affected UL, all the children in the present study stated that they can now use both hands to do activities of daily living that they could not do before, and that they often use both hands and are unaware that they are using the affected hand. Some comments allude to the discovery of the affected hand, which they did not perceive before, or to having fun with it, although it does not work like the other hand. This suggests an increase in affected hand spontaneous use in unimanual and bimanual activities without excessive dominance from the unaffected hand, perhaps due to greater representation at the cortical level [26,27]. Thus, neuroplastic cortical changes in children following mCIMT, which have been studied [28] using functional magnetic resonance imaging (fMRI), have shown increases in the cortical activation of the sensorimotor cortex contralateral to the affected hand, rather than increases in the activation of the contralateral motor cortex. Since this feedback is necessary for perceptual awareness of movement, mCIMT may contribute to increased affected hand use through the reduction/resolution of inattention to contralesional space, or hemispatial neglect, reducing the “development disregard” [28,29]. The repetition of activities is crucial to obtain different strategies and better spontaneous use, and all the children enjoyed the execution of the activities, since they were motivated. It was known to strike a balance between the demands of the task and the child’s ability to avoid frustration and achieve an intrinsic motivation that would allow continuity and enjoyment in the proposed activities [27], in addition to sharing time with their parents, which increased their desire to maintain the activity.

The execution of the activities was also successful due to the parent’s involvement and their training in the intervention through the therapist. As stated, after finishing the training, all of them felt comfortable and confident with the execution, with the follow-up carried out every week being essential to maintaining adherence to the intervention. Interventions typically involve the child’s parents and are aimed at promoting physical and psychological well-being through improving their capacity to adapt to and manage condition-related challenges. Family-focused interventions have been further differentiated as being either psychoeducational or relationship-focused [30]. Reviews on family-focused intervention research have provided evidence of their efficacy with regard to improving condition management and adherence, as well as family or family member functioning [30,31,32,33].

The natural environment (home) offered a comfortable and enriched place to encourage learning during modified constraint-induced movement therapy practice for children and their parents. Furthermore, the training schedule can be tailored to fit the family’s daily routine. A home-based intervention can also save the family time and money in terms of commuting, and parents can be more involved throughout the process, increasing opportunities for parent–child interaction [30]. In addition to the influence of the environment, in this case, the home, the adaptation of the protocol, using a daily dose of 2 h distributed over two periods, facilitates execution and increases adherence, making the intervention possible [34].

The feeling of not wanting to end the intervention on the part of the parents who conducted the intervention demonstrates their satisfaction with helping their children to obtain visible benefits after the intervention and, as parents, being aware of the limitations of their children and how they turn into functionality. When the quality of life of children with cerebral palsy is assessed, parents score lower than children [35], since they think about quality, and making the intervention at home through their involvement could help them to observe the actual functioning of their children and be more objective when observing the quality of life of the latter.

Regarding the limitations, it must be pointed out that this is a case series study, in which the children had a variety of ages, although the responses were similar, which suggests that children under 4 years of age and from 9 years of age and adolescents may demonstrate different behavior in the implementation, and the experiences of parents and their children (in these age ranges) should be collected to compare the similarities and differences with this research and study the influence of age on the experience with the application of mCIMT.

The findings obtained in the present study about the experiences of children and the parents who were involved in the mCIMT, the type of protocol applied at home, the use of containment of the unaffected hand, and the selection of recreational activities based on children’s motivation and needs, would allow the following:

▪ Improving the practice of the intervention in the natural environment (home)
▪ Favoring a protocol adapted to the needs and worries of parents to make its execution possible at home.
▪ Empowering parents to allow therapy to take place appropriately.
▪ Giving satisfaction and fostering the bond between the parents and children and the therapist.
▪ Guaranteeing adaptation to therapy through continuous follow-up and feedback.

This would promote a better practice of professionals and, consequently, an improvement through intervention focused on the family, allowing us to know the barriers and facilitators to promote the strengths of the child and parents and address the limitations to turn them into improvement strategies, thus, favoring the participation of the children and their families within their natural context, i.e., their home.

## 5. Conclusions

A facilitator within mCIMT would be the participation of the child’s parents through previous training and its approach at home, allowing greater family satisfaction and child–parent–therapist interaction to avoid complications. Containment use in the unaffected hand would not be a barrier. The barrier would be the affected hand use for the execution of unimanual activities, and, thus, motivation must be ensured, through simple and playful activities, in order to avoid frustration in the use.

## Figures and Tables

**Table 1:** Questions asked to the parent involved in the intervention and the children who performed 50 h modified constraint-induced movement therapy (mCIMT) at home.

**Main Open-Ended Questions for Children and Parents**	
**Children**	**Parents**
How did you feel during the application of this therapy?	What was your experience from this therapy? What was your experience during the mCIMT application? Were your expectations met?
**Specific Questions for Children and Parents**	
**Children**	**Parents**
What did you feel when they covered your hand with the bandage so that you could use your “less-used” hand? Was it easy to wear the containment? Did you want to take it off? Did you feel bad or want to cry? Was it more difficult to wear the bandage or use the “less-used” hand? Were you comfortable with the use of containment during the activities? What did doing these exercises at home mean to you? Did you have a good time with mom or dad? Would you have preferred to go to your therapist? Would you repeat the activities? Were the activities to your liking or were you bored? How was the session with mom or dad? What did you learn when you used your “less-used” hand?	Can you tell me about your family? How old is your child? Can you tell me about other family members? What is the usual behavior of the child during therapy? Did the child try to take the containment off during the therapy? Did the child regularly wear a recommended containment for a recommended amount of time? Was it difficult to perform the protocol for 2 h per day for 5 weeks? Was it more difficult to do the therapy dose or the therapy at home? How is CIMT different from other therapies? Do you prefer home therapy or not? What did having a follow-up per week with a therapist mean to you? Was it important to obtain this help to complete the therapy? Are you satisfied with the results of CIMT for your child’s upper limb function progress? What was your feeling when the therapy finished?

**Table 2:** Characteristics of the participants (children and parents who conducted the intervention).

P	A	SEX	G. W	Lesion	MACS	GMFCS	Hemip. Side	Add. Impairs	Parent	Parent’s Status	Parent’s Education
**Ch1**	4	F	Term	PS	II	I	Left	No	Mother	Married	High Sch.
**Ch2**	5	M	Term	PS	II	I	Right	No	Mother	Married	High Sch.
**Ch3**	5	M	Preterm	PS	II	I	Left	No	Mother	Married	Elem. Sch.
**Ch4**	5	M	Preterm	PS	II	I	Left	No	Mother	Married	Elem. Sch.
**Ch5**	7	F	Term	PS	II	I	Right	Speech	Mother	Divorced	Elem. Sch.
**Ch6**	6	F	Term	PS	II	I	Right	Speech	Mother	Married	High Sch.
**Ch7**	8	F	Preterm	PS	II	I	Left	Speech	Father	Divorced	High Sch.
**Ch8**	8	M	Term	PS	II	I	Left	No	Mother	Married	Elem. Sch.

P: Participants; Ch: Child; A: Age; Sex: F: Female; M: Male. G. W: Gestational Week: preterm (>32 weeks and <36 weeks), term: >36 weeks; PS: Perinatal Stroke; MACS: Manual Ability Classification System; GMFCS: Gross Motor Function Classification System. Hemip. side: Hemiplegic side; Add. impairs: Added impairments. Parent: Parent who conducted the intervention at home; Elem. Sch: Elementary school; Sch: School.

## References

[1] Sakzewski L., Ziviani J., Boyd R. (2009). Systematic review and metaanalysis of therapeutic management of upper-limb dysfunction in children with congenital hemiplegia. Pediatrics.

[2] Morris D.M., Taub E., Mark V.W. (2006). Constraint-induced movement therapy: Characterizing the intervention protocol. Eur. Med..

[3] Charles J., Gordon A.M. (2005). A critical review of constraint-induced movement therapy and forced use in children with hemiplegia. Neural Plast..

[4] Hoare B., Imms C., Carey L., Wasiak J. (2007). Constraint-induced movement therapy in the treatment of the upper limb in children with hemiplegic cerebral palsy: A Cochrane systematic review. Clin. Rehabil..

[5] Hoare B.J., Wallen M.A., Thorley M.N., Jackman M.L., Carey L.M., Imms C. (2019). Constraint-induced movement therapy in children with unilateral cerebral palsy. Cochrane Database Syst. Rev..

[6] Ilieva E., Ilieva A. (2020). What is the effect of constraint-induced movement therapy on children with unilateral cerebral palsy? A Cochrane Review summary with commentary. Dev. Med. Child Neurol..

[7] Gordon A.M., Hung Y.C., Brandao M., Ferre C.L., Kuo H.C., Friel K., Petra E., Chinnan A., Charles J.R. (2011). Bimanual training and constraint-induced movement therapy in children with hemiplegic cerebral palsy: A randomized trial. Neurorehabilit. Neural Repair.

[8] Sakzewski L., Ziviani J., Abbott D.F., Macdonell R.A., Jackson G.D., Boyd R.N. (2011). Randomized trial of constraint-induced movement therapy and bimanual training on activity outcomes for children with congenital hemiplegia. Dev. Med. Child Neurol..

[9] Eliasson A.C., Shaw K., Berg E., Krumlinde-Sundholm L. (2011). An ecological approach of Constraint Induced Movement Therapy for 2-3-year-old children: A randomized control trial. Res. Dev. Disabil..

[10] Al-Oraibi S., Eliasson A.C. (2011). Implementation of constraint-induced movement therapy for young children with unilateral cerebral palsy in Jordan: A home-based model. Disabil. Rehabil..

[11] Huang W.C., Chen Y.J., Chien C.L., Kashima H., Lin K.C. (2011). Constraint-induced movement therapy as a paradigm of translational research in neurorehabilitation: Reviews and prospects. Am. J. Transl. Res..

[12] Peurala S.H., Kantanen M.P., Sjögren T., Paltamaa J., Karhula M., Heinonen A. (2012). Effectiveness of constraint-induced movement therapy on activity and participation after stroke: A systematic review and meta-analysis of randomized controlled trials. Clin. Rehabil..

[13] Gilmore R., Ziviani J., Sakzewski L., Shields N., Boyd R. (2010). A balancing act: Children’s experience of modified constraint-induced movement therapy. Dev. Neurorehabil..

[14] Mancini M.C., Brandão M.B., Dupin A., Drummond A.F., Chagas P.S., Assis M.G. (2013). How do children and caregivers perceive their experience of undergoing the CIMT protocol?. Scand. J. Occup. Ther..

[15] Manzoor N., Kashif M., Haroon B., Dastgir A., Iram H. (2019). Parent’s perception of constraint induced movement therapy in cerebral palsy management in rehabilitation centers of Lahore. J. Pak. Med. Assoc..

[16] Scime N.V., Bartlett D.J., Brunton L.K., Palisano R.J. (2017). Parents’ Experiences and Perceptions when Classifying their Children with Cerebral Palsy: Recommendations for Service Providers. Phys. Occup. Ther. Pediatr..

[17] Palomo-Carrión R., Romero-Galisteo R.P., Pinero-Pinto E., López-Muñoz P., Romay-Barrero H., José F.G.S. (2020). Application of Low-Intensity Modified Constraint-Induced Movement Therapy to Improve the Affected Upper Limb Functionality in Infantile Hemiplegia with Moderate Manual Ability. Case Ser. Child..

[18] McConnell K., Johnston L., Kerr C. (2014). Efficacy and acceptability of reduced intensity constraint-induced movement therapy for children aged 9-11 years with hemiplegic cerebral palsy: A pilot study. Phys. Occup. Ther. Pediatr..

[19] Graneheim U.H., Lundman B. (2004). Qualitative content analysis in nursing research: Concepts, procedures and measure to achieve trustworthiness. Nurse Educ. Today.

[20] DeLuca S.C., Echols K., Charles R.L., Ramey S.L. (2006). Intensive pediatric constraint-induced therapy for children with cerebral palsy: Randomized, controlled, crossover trial. J. Child Neurol..

[21] Gordon A.M., Charles J., Wolf S.L. (2005). Methods of constraint induced movement therapy for children with hemiplegic cerebral palsy: Development of a child-friendly intervention for improving upper-extremity function. Arch. Phys. Med. Rehabil..

[22] Poulsen A.A., Rodger S., Ziviani J.M. (2006). Understanding children’s motivation from a self-determination theoretical perspective: Implications for practice. Aust. Occup. Ther. J..

[23] Deci E.L., Ryan R.M. (2008). Facilitating optimal motivation and psychological well-being across life’s domains. Can. Psychol..

[24] Miller L., Ziviani J., Ware R.S., Boyd R.N. (2014). Mastery Motivation as a predictor of occupational performance following upper limb intervention for school-aged children with congenital hemiplegia. Dev. Med. Child Neurol..

[25] Aarts P.B., Jongerius P.H., Geerdink Y.A., van Limbeek J., Geurts A.C. (2010). Effectiveness of modified constraint-induced movement therapy in children with unilateral spastic cerebral palsy: A randomized controlled trial. Neurorehabil. Neural Repair.

[26] Taub E., Miller N.E., Novack T.A., Cook E.W., Fleming W.C., Nepomuceno C.S., Connell J.S., Crago J.E. (1993). Technique to improve chronic motor deficit after stroke. Arch. Phys. Med. Rehabil..

[27] Csikszentmihalyi M. (1990). Flow: The Psychology of Optimal Experience.

[28] Sutcliffe T.L., Gaetz W.C., Logan W.J., Cheyne D.O., Fehlings D.L. (2007). Cortical reorganization after modified constraint-induced movement therapy in pediatric hemiplegic cerebral palsy. J. Child Neurol..

[29] Brady K., Garcia T. (2009). Constraint-induced movement therapy (CIMT): Pediatric applications. Dev. Disabil. Res. Rev..

[30] Chesla K. (2010). Do family interventions improve health?. J. Fam. Nurs..

[31] Carr D., Springer K. (2010). Advances in families and health research in the 21st century. J. Marriage Fam..

[32] Champaloux S., Young D. (2015). Childhood chronic health conditions and educational attainment: A social ecological approach. J. Adolesc. Health.

[33] Brusco N.K., Taylor N.F., Watts J.J., Shields N. (2014). Economic evaluation of adult rehabilitation: A systematic review and meta-analysis of randomized controlled trials in a variety of settings. Arch. Phys. Med. Rehabil..

[34] Ferre C.L., Gordon A.M. (2017). Coaction of individual and environmental factors: A review of intensive therapy paradigms for children with unilateral spastic cerebral palsy. Dev. Med. Child Neurol..

[35] Varni J., Limbers C. (2009). The Pediatric Quality of Life Inventory: Measuring Pediatric Health-Related Quality of Life from the Perspective of Children and Their Parents. Pediatr. Clin. N. Am..

